# Non-Coding RNAs as Emerging Regulators in Kidney Pathophysiology: From Molecular Mechanisms to Therapeutic Potential

**DOI:** 10.3390/genes16111328

**Published:** 2025-11-03

**Authors:** Petar Todorović, Nikola Pavlović, Mirko Maglica, Patricija Bajt, Nela Kelam, Fila Raguž, Katarina Vukojević

**Affiliations:** 1 Department of Anatomy, Histology and Embryology, University of Split School of Medicine, 21000 Split, Croatia; petar.todorovic@mefst.hr (P.T.); patricija.bajt@mefst.hr (P.B.); nela.kelam@mefst.hr (N.K.); 2 Department of Pathophysiology, University of Split School of Medicine, 21000 Split, Croatia; nikola.pavlovic@mefst.hr; 3 Department of Anatomy, School of Medicine, University of Mostar, 88000 Mostar, Bosnia and Herzegovina; mirko.maglica@mef.sum.ba; 4 Department of Nephrology, University Clinical Hospital Mostar, 88000 Mostar, Bosnia and Herzegovina; fila.raguz@mef.sum.ba; 5 Mediterranean Institute for Life Sciences, University of Split, 21000 Split, Croatia; 6 Center for Translational Research in Biomedicine, University of Split School of Medicine, 21000 Split, Croatia

**Keywords:** non-coding RNAs, microRNAs, long non-coding RNAs, circular RNAs, kidney disease, biomarkers, fibrosis, therapeutic targeting

## Abstract

The kidney’s intricate physiology relies on finely tuned gene regulatory networks that coordinate cellular responses to metabolic, inflammatory, and fibrotic stress. Beyond protein-coding transcripts, non-coding RNAs (ncRNAs), including microRNAs (miRNAs), long non-coding RNAs (lncRNAs), and circular RNAs (circRNAs), have emerged as pivotal regulators of renal biology. By modulating transcriptional, post-transcriptional, and epigenetic pathways, ncRNAs govern podocyte integrity, tubular adaptation, intercellular signaling, and immune activation. Dysregulation of these networks is now recognized as a hallmark of major kidney diseases, ranging from diabetic nephropathy and acute kidney injury to chronic kidney disease, glomerulopathies, and polycystic kidney disease. Mechanistic studies have revealed how pathogenic ncRNAs drive apoptosis, inflammation, fibrosis, and cystic remodeling, while protective ncRNAs mitigate these processes, highlighting their dual roles as both disease mediators and therapeutic targets. The exceptional stability of ncRNAs in urine, plasma, and exosomes further positions them as minimally invasive biomarkers with diagnostic and prognostic value. Translational advances include anti-miR and mimic-based therapies (e.g., lademirsen targeting miR-21, miR-29 mimics, anti-miR-17 oligonucleotides), alongside lncRNA silencing strategies, although challenges in delivery, safety, and redundancy remain significant. This review integrates molecular mechanisms with translational perspectives, providing a comprehensive synthesis of how ncRNAs shape renal pathophysiology. By bridging mechanistic insights with emerging diagnostic and therapeutic applications, we highlight the potential of ncRNAs to transform nephrology, paving the way for biomarker-driven precision medicine and novel interventions aimed at intercepting kidney injury at its regulatory roots. In clinical terms, ncRNA-based biomarkers and therapeutics promise earlier detection, more precise risk stratification, and individualized treatment selection within precision nephrology.

## 1. Introduction

The last two decades have seen a paradigm shift in molecular biology with the discovery and characterisation of non-coding RNAs (ncRNAs) as essential regulators of gene expression. In addition to protein-coding transcripts, ncRNAs, including microRNAs (miRNAs), long non-coding RNAs (lncRNAs), and circular RNAs (circRNAs), serve as important modulators of cellular processes [1,2]. Their diverse molecular mechanisms and intricate regulatory networks are increasingly recognised in the context of renal biology and disease. This review aims to provide a comprehensive and integrative overview of these different classes of molecules, their role in renal cell biology, their involvement in major renal diseases, and their emerging potential as biomarkers and therapeutic targets [3,4,5].

The unique complexity of the kidney lies in its highly specialised and metabolically active cellular composition, which includes podocytes, tubular epithelial cells, and other renal cell types that interact dynamically to maintain organ function [6]. This heterogeneity creates a complex regulatory landscape in which ncRNAs fine-tune cellular phenotypes and responses and mediate processes such as inflammation, fibrosis, and metabolic adaptation [7]. These pathological processes are central to various kidney diseases such as diabetic nephropathy, acute kidney injury (AKI), chronic kidney disease (CKD) with fibrosis, and glomerulonephritis. Understanding the ncRNA networks within specific renal cell populations is crucial for deciphering disease mechanisms and progression [8,9,10,11,12,13].

**Figure 1:**
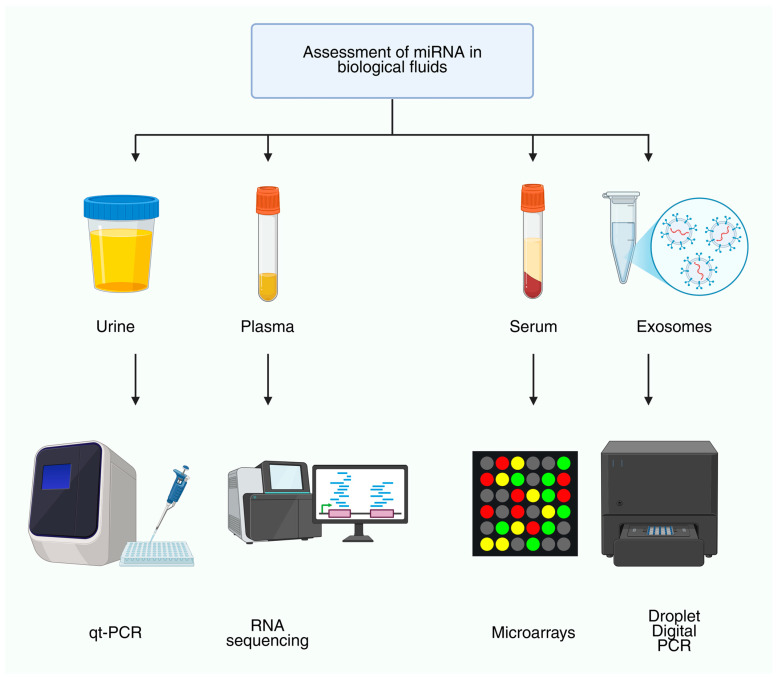
Assessment of miRNAs in biological fluids. Overview of commonly sampled matrices (urine, plasma, serum, exosomes) and analytical platforms used for quantification (qRT-PCR, RNA sequencing, microarrays, droplet digital PCR). This workflow highlights pre-analytical diversity across fluids and platform-dependent sensitivity/precision considerations that influence biomarker development.

Our review systematically addresses the major molecular classes of ncRNAs, including miRNAs, lncRNAs and circRNAs, focusing on their biogenesis, functions and interactions, as well as their roles in renal cell types and disease mechanisms. We highlight ncRNA dysregulation across kidney diseases, emphasizing mechanistic insights, disease-specific signatures and translational perspectives. Circulating ncRNAs as minimally invasive biomarkers and emerging therapeutic strategies, including antagomirs, mimics and lncRNA silencing, are discussed together with current challenges and future directions for integrating ncRNA biology into precision nephrology (Figure 1). By focusing on circRNAs, renal cell specificity and clinical applications, this review provides a concise and comprehensive synthesis of recent advances in molecular nephrology. While we synthesize recent advances across mechanisms and clinical translation, several methodological limitations still shape how these findings should be interpreted. Despite rapid advances in ncRNA research, several technical limitations still constrain the field and complicate translation. Technical challenges, particularly the lack of robust and standardized methodologies for ncRNA detection and quantification, represent a major barrier to clinical implementation. Variability in sample collection, RNA extraction, normalization and data analysis can produce inconsistent results and limit reproducibility across laboratories [13,14]. Moreover, the low abundance and natural instability of certain ncRNAs, such as lncRNAs, further complicate accurate measurement [15]. Addressing these issues requires the development and adoption of sensitive and standardized detection methods, the use of shared reference materials and rigorous bioinformatics pipelines to ensure that results are comparable and clinically meaningful [16]. Establishing such methodological consistency is essential before ncRNAs can be reliably integrated as biomarkers or therapeutic targets in nephrology. Figure 1 illustrates the commonly sampled biofluids (urine, plasma, serum, exosomes) and the principal analytical platforms used for miRNA quantification (qRT-PCR, RNA sequencing, microarrays, droplet digital PCR), providing a concise methodological overview relevant to biomarker development.

In parallel with bulk profiling, single-cell RNA sequencing (scRNA-seq) and spatial transcriptomics are increasingly applied to the kidney to resolve ncRNA programs at cellular and anatomical resolution. scRNA-seq atlases in mouse and human kidney delineate fine cell-type heterogeneity and injury-state transitions, enabling assignment of ncRNA signatures to specific nephron segments and immune niches [17,18]. Spatially resolved transcriptomics further localizes these signals in situ, mapping fibrosis and tubular–interstitial microenvironments and revealing injury-specific neighborhoods not captured by bulk assays [19,20]. Emerging single-cell resources also chart cell- and age-specific long non-coding RNA expression in kidney, expanding candidate regulators for mechanistic study [12]. Together, these technologies refine disease mechanisms and nominate more precise ncRNA biomarkers and therapeutic targets for nephron-level precision medicine [21].

## 2. Literature Search Strategy

Relevant studies published over the last 10 years were identified through a PubMed/MEDLINE search (accessed on 17 July 2025) using combinations of the following keywords: non-coding RNAs, microRNAs, long non-coding RNAs, circular RNAs, kidney disease, diabetic nephropathy, acute kidney injury, chronic kidney disease, glomerulonephritis, polycystic kidney disease, biomarkers, and therapeutics. Clinical trial data were additionally retrieved from ClinicalTrials.gov and the EU Clinical Trials Register (accessed on 17 April 2025). We emphasized original research articles, clinical trials, cohort studies, and mechanistic experimental work.

Systematic reviews and meta-analyses were not the primary focus but were selectively included when they provided essential background or summarized landmark findings. Only English-language articles were included. Titles and abstracts were screened for relevance, and full-text articles were reviewed to extract information directly related to the roles of ncRNAs in kidney pathophysiology, biomarker development, and therapeutic targeting. To ensure completeness, complementary searches were performed in Web of Science, Scopus, and Google Scholar.

**Figure 2:**
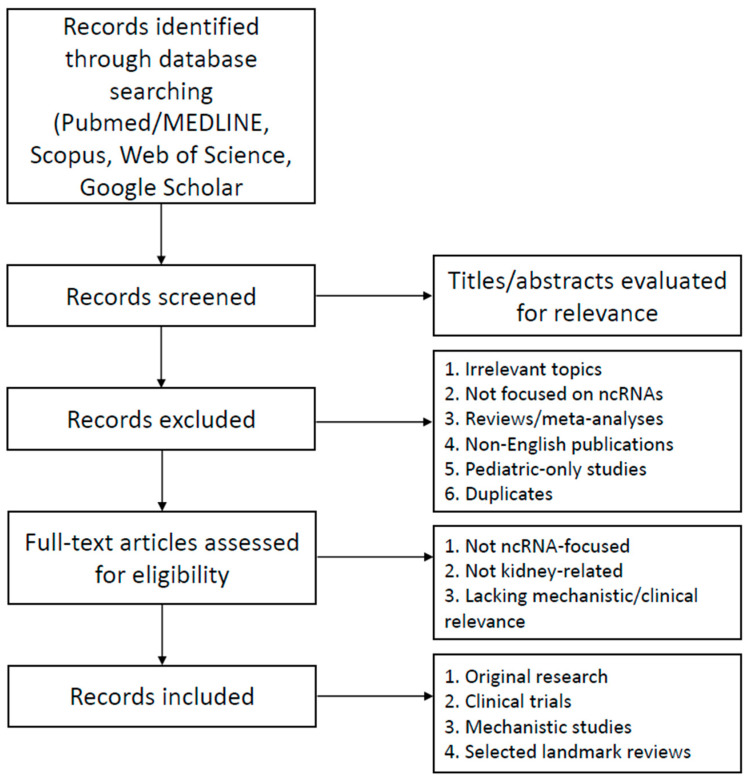
Flowchart of the literature search and selection process. Records were identified through PubMed/MEDLINE, Scopus, Web of Science, and Google Scholar. After screening of titles and abstracts, irrelevant studies, non-ncRNA-focused articles, reviews/meta-analyses, non-English publications, pediatric-only studies, and duplicates were excluded. Full-text articles were then assessed for eligibility, and those not related to ncRNAs, not kidney-focused, or lacking mechanistic/clinical relevance were excluded. The final selection included original research articles, clinical trials, mechanistic studies, and selected landmark reviews.

Inclusion criteria were original research studies, experimental and translational work, clinical trials, and highly relevant reviews addressing microRNAs, long non-coding RNAs, or circular RNAs in kidney physiology and disease. Both animal and human studies were considered. Exclusion criteria included non-English publications, conference abstracts without full data, editorials, pediatric-only studies, and studies not focused on ncRNAs or not related to kidney pathology. The overall search, screening, and selection process is summarized in Figure 2.

## 3. Molecular Classes of Non-Coding RNAs

**Figure 3:**
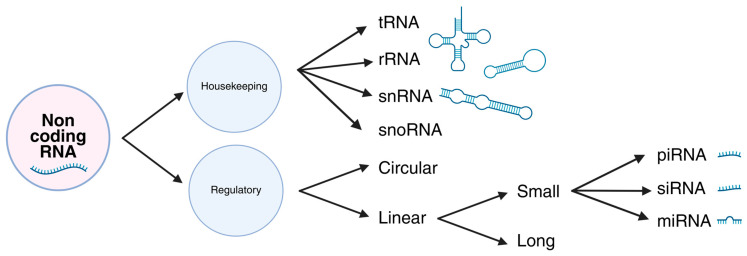
Molecular classes of non-coding RNAs (ncRNAs). NcRNAs are broadly divided into housekeeping ncRNAs (e.g., tRNAs, rRNAs, snRNAs, snoRNAs), which are constitutively expressed and essential for basal cellular functions, and regulatory ncRNAs, which control gene expression. Regulatory ncRNAs include linear forms, such as microRNAs (miRNAs) and long non-coding RNAs (lncRNAs), as well as other small RNAs (siRNAs, piRNAs), and circular RNAs (circRNAs), which are generated by back-splicing. Together, these classes form interconnected regulatory networks that influence cellular homeostasis and disease processes.

Non-coding RNAs (ncRNAs) are a diverse group of RNA molecules that do not encode proteins but play essential roles in regulating gene expression and other cellular processes through multiple mechanisms. They can be divided into housekeeping RNAs, such as transfer RNAs and ribosomal RNAs, which support fundamental cellular functions, and regulatory RNAs, which modulate gene expression. Regulatory RNAs include linear RNAs and circular RNAs (circRNAs). Linear RNAs are further divided into short RNAs, such as microRNAs (miRNAs) and long non-coding RNAs (lncRNAs) [1,22]. The main classes of ncRNAs are summarized in Figure 3.

Among the numerous classes of ncRNAs, miRNAs, lncRNAs and circRNAs have attracted particular attention in nephrology because of their important roles in kidney cell biology and disease [23,24]. miRNAs are small, single-stranded RNA molecules approximately 20–24 nucleotides in length that primarily regulate gene expression at the post-transcriptional level [25]. They are incorporated into the RNA-induced silencing complex (RISC), which guides them to complementary sequences, usually located in the 3′ untranslated regions (3′-UTRs) of target messenger RNAs. Upon AGO–RISC binding to the 3′-UTR, GW182 recruits the CCR4–NOT complex to deadenylate and decap the transcript, leading predominantly to mRNA destabilization, with additional inhibition of translation initiation [26,27]. Binding to these sequences results in translational repression or mRNA degradation [28,29]. Because a single miRNA can target multiple mRNAs, it can broadly regulate cellular networks. Additionally, miRNAs are highly conserved across species and often show tissue-specific expression, highlighting their precise regulatory roles [30]. Their stability in biofluids, particularly when enclosed in exosomes, along with their short length and high specificity, emphasizes their strong potential for clinical applications [31]. lncRNAs, in contrast, are heterogeneous transcripts longer than 200 nucleotides.

They regulate gene expression through multiple mechanisms by interacting with DNA, mRNA, and proteins, affecting transcriptional, post-transcriptional, and epigenetic processes [32]. In the nucleus, lncRNAs can bind specific genomic loci and recruit chromatin-modifying complexes or transcription factors, modulating chromatin, epigenetic marks, and gene transcription. In the cytoplasm, they interact with mRNAs and miRNAs, either functioning as molecular sponges that bind and inactivate miRNAs, or pairing with mRNAs to regulate their splicing, stability, and translation [3,33]. Furthermore, lncRNAs are dynamic regulators whose activity depends on the cellular environment, allowing them to coordinate signals and modulate cell function [34].

circRNAs are single-stranded RNAs with a covalently closed circular structure, formed by back-splicing of pre-mRNA. This circular structure makes them resistant to exonuclease-mediated degradation, giving them high molecular stability. circRNAs can interact with miRNAs, acting as molecular sponges, or bind RNA-binding proteins to modulate transcriptional and post-transcriptional processes [35]. They are conserved across species and show dynamic, tissue-specific expression [36]. Their stability makes them particularly promising for clinical applications, including reliable detection in biofluids such as urine or plasma, as well as their potential use as biomarkers and therapeutic targets [37].

Together, miRNAs, lncRNAs, and circRNAs work together in connected networks to control gene expression, with each type having a unique and complementary role. Understanding their structure and mechanisms provides a strong foundation for exploring kidney-specific functions, disease associations, and potential diagnostic and therapeutic applications [23].

In addition to these major classes, PIWI-interacting RNAs (piRNAs) are 24–32-nt small RNAs that bind PIWI proteins to mediate transcriptional and post-transcriptional silencing, best characterized in the germline but increasingly reported in somatic tissues [38]. Emerging kidney-relevant data include clear cell renal cell carcinoma, where piR-57125 suppresses metastasis by targeting CCL3 and modulating AKT/ERK signaling, suggesting disease relevance in renal tissue. Given the early stage of renal piRNA research, we note their potential while focusing this review on miRNAs, lncRNAs and circRNAs [39]. Small nucleolar RNAs (snoRNAs), typically 60–300 nucleotides, guide site-specific 2′-O-methylation and pseudouridylation of rRNA and other RNAs, and several have emerging relevance in renal disease. In diabetic kidney disease, circulating snoRNAs measured by small-RNA sequencing associate with DKD status and renal traits, extending biomarker candidates beyond miRNAs [40]. In acute kidney injury, Snord3a is upregulated in renal tubules and drives injury progression by promoting STING-linked ferroptosis; genetic or antisense inhibition of Snord3a alleviates tubular damage in mouse AKI models, nominating snoRNAs as mechanistic targets [41]. In oncology-related kidney pathology, urinary extracellular-vesicle snoRNAs serve as non-invasive biomarkers for clear cell renal cell carcinoma, with several snoRNAs showing diagnostic performance in patient cohorts [42]. While the renal piRNAs and snoRNA field is earlier-stage than miRNAs, lncRNAs and circRNAs, these data support context-dependent roles in injury, immunity and biomarker development, and we therefore briefly note snoRNAs here while focusing the review on the three major classes.

## 4. ncRNAs in Renal Cell Biology

Non-coding RNAs (ncRNAs), including microRNAs (miRNAs), long non-coding RNAs (lncRNAs), and circular RNAs (circRNAs), are increasingly recognized as pivotal modulators of renal cell biology, critically shaping the function and fate of key cell types such as podocytes and tubular epithelial cells, and representing both mechanistic insights and therapeutic avenues [1]. Within podocytes, lncRNAs exert profound regulation of cell survival and injury responses. Under diabetic or fibrotic stress, several lncRNAs show increased expression, including CASC15, HOTAIR, PVT1, PRINS, MALAT1, GM15645, GM5524 and LOC105374325, which converge on apoptosis, autophagy and profibrotic cascades, whereas LINC01619 is decreased, consistent with worsened ER-stress signaling [43]. For example, high-glucose exposure elevates CASC15, which sponges miR-43c to promote podocyte apoptosis [44], while HOTAIR is modulated by NF-κB–p65 and may regulate adjacent HOXC11 in CKD [45]. Similarly, PVT1 and PRINS contribute to podocyte injury via TGF-β1/FN1 accumulation and Smad7-dependent pathways, respectively; conversely, decreased LINC01619 correlates with worse diabetic nephropathy, acting through miR-27a and ER stress [46,47]. MALAT1 expression is upregulated in response to high-glucose conditions, where it interferes with β-catenin feedback loops [48], while GM15645, GM5524, and LOC105374325 enhance apoptosis and autophagy by modulating Bcl2, caspase-3, Bax, LC3, and other key regulators [49,50].

In renal tubular epithelial cells, particularly under diabetic nephropathy, circRNAs emerge as significant factors. Several circRNAs including circ_0060077 [51], circ_0068087 [52], circ_TAOK1 [53], circ_0003928, and hsa_circ_0003928 are upregulated in peripheral blood and high-glucose-treated HK-2 cells [54]. These circRNAs modulate miRNA-target networks, e.g., circs sponging miR-145-5p, miR-106a-5p, miR-142-3p to regulate VASN, ROCK2, SOX6, as well as miR-31-5p or miR-151-3p affecting MAPK6 and Anxa2—thereby controlling apoptosis, oxidative stress, inflammation, and fibrotic markers such as Bcl-2, Bax, TNF-α, IL-6, IL-1β, FN, collagen-I/IV, and SOD activity [51,53,54].

Circular RNAs themselves are distinguished from linear ncRNAs by their covalently closed loop structure, which lacks 5′ caps and poly-A tails, conferring exceptional stability and resistance to exonucleases. CircRNAs are generated by back-splicing or lariat-driven circularization and can originate from exonic (EcircRNA), intronic (ciRNA), or exon-intron (EIciRNA) sequences. They are localized in the nucleus, cytoplasm, or secreted in exosomes, and function through diverse mechanisms: acting as miRNA or protein sponges, scaffolding transcriptional complexes, regulating transcription or translation, and even encoding peptides via IRES-driven translation in select instances [55].

In the kidney context, circRNAs have been implicated across a spectrum of pathologies: acute kidney injury, diabetic nephropathy, glomerular diseases, hypertensive nephropathy, allograft rejection, chronic kidney disease complications, and renal cancer. Their stability and presence in extracellular vesicles make them promising diagnostic and prognostic biomarkers, as well as potential therapeutic targets [56,57,58].

Taken together, ncRNAs act as central regulators of key signaling networks that maintain or disrupt renal cell homeostasis. Across both podocytes and tubular epithelial cells, diverse ncRNA classes converge on TGF-β/Smad, NF-κB, β-catenin, and MAPK pathways, as well as core apoptotic (Bcl-2, Bax, caspase-3) and fibrotic (FN1, collagen I/IV) cascades. lncRNAs primarily shape stress and transcriptional programs, circRNAs fine-tune miRNA–mRNA interaction networks, and miRNAs mediate post-transcriptional control, collectively forming interconnected layers that regulate apoptosis, autophagy, inflammation, and extracellular matrix turnover. These shared regulatory axes underscore the network-level integration of ncRNAs in renal cell biology and provide a mechanistic basis for their translational potential as biomarkers and therapeutic targets.

## 5. ncRNAs in Metabolic and Acute Renal Injury

**Figure 4:**
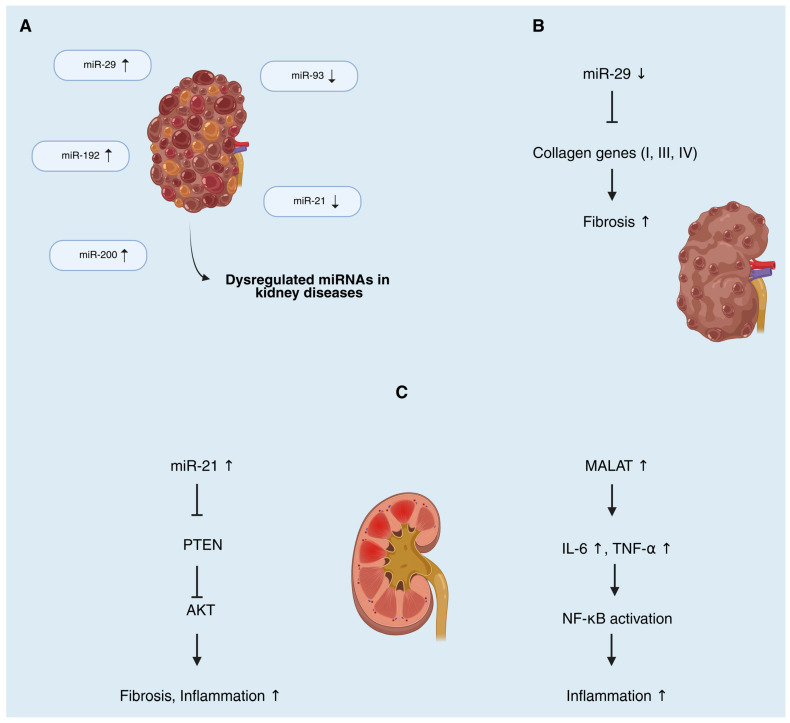
Representative examples of dysregulated ncRNAs in kidney diseases. (**A**) Several microRNAs are altered in renal pathologies, including increased expression of miR-29, miR-192, and miR-200, and decreased expression of miR-21 and miR-93. (**B**) Downregulation of the anti-fibrotic miR-29 family leads to derepression of collagen genes (types I, III, IV), thereby promoting renal fibrosis. (**C**) Upregulation of miR-21 suppresses PTEN, activates AKT signaling, and drives inflammation and fibrosis. In parallel, elevated lncRNA MALAT1 enhances IL-6 and TNF-α production, leading to NF-κB activation and exacerbated inflammation. Arrows indicate the direction of expression change: upward arrows (↑) represent increased expression, whereas downward arrows (↓) denote decreased expression in kidney disease samples compared with controls.

Non-coding RNAs (ncRNAs), notably microRNAs (miRNAs), long non-coding RNAs (lncRNAs), and circular RNAs (circRNAs), have emerged as critical regulators across renal pathologies [59]. They orchestrate gene expression networks that drive inflammation, fibrosis, and cellular injury in kidney disease, and their disease-specific expression patterns make them attractive as biomarkers and therapeutic targets [59,60]. Representative examples of dysregulated ncRNAs and their mechanistic consequences in kidney disease are illustrated in Figure 4.

Multiple ncRNAs contribute to the development and progression of DN. Among miRNAs, miR-21 is consistently upregulated in diabetic kidneys and in circulation, where it promotes epithelial-to-mesenchymal transition, extracellular matrix deposition, inflammation, and fibrosis by targeting pathways like PTEN/AKT, TGF-β/SMAD, and NF-κB [61]. Notably, miR-21 has been identified as a pivotal pathogenic factor in DN and is being explored as a diagnostic and prognostic biomarker [61]. Importantly, the actions of miR-21 and miR-29 are context-dependent. In chronic metabolic injury (DN), sustained tubular/interstitial miR-21 elevation is profibrotic, whereas transient induction during acute stress in selected compartments can exert anti-apoptotic or cytoprotective effects [62,63]. Conversely, the miR-29 family functions as an antifibrotic brake in DN by restraining collagen programs, yet early stress responses can transiently suppress miR-29, permitting short-term matrix remodeling before re-establishment of homeostasis [64]. Cell type (e.g., tubular vs. endothelial vs. podocyte) and disease stage (early vs. established fibrosis) critically shape these outcomes [64]. Several lncRNAs are dysregulated in DN and modulate these miRNA-mediated pathways. For example, lncRNA TUG1 (taurine upregulated gene 1) is downregulated in DN, leading to mitochondrial dysfunction in podocytes; transgenic overexpression of TUG1 in podocytes of diabetic mice preserved mitochondrial biogenesis via PGC-1α and protected against nephropathy [65]. In contrast, the X-inactive specific transcript lncRNA XIST is markedly upregulated in DN and sponges the anti-fibrotic miR-93-5p, thereby de-repressing Cyclin-Dependent Kinase Inhibitor 1A (CDKN1A) and exacerbating renal fibrosis [65]. Silencing XIST in diabetic models restored miR-93 activity and attenuated interstitial fibrosis, highlighting XIST as a potential therapeutic target [65,66]. circRNAs are also implicated in DN: dozens of circRNAs show altered expression in diabetic kidneys, functioning as miRNA “sponges.” For instance, circEIF4G2 is significantly upregulated in tubuloepithelial cells under high glucose and aggravates renal fibrosis by sequestering miR-218, leading to increased SERBP1 and downstream TGF-β1/Collagen I production [67]. Such findings underscore the biomarker potential of ncRNAs in DN—e.g., circulating levels of certain lncRNAs (ARAP1-AS1/AS2) and circRNAs have been proposed as novel minimally invasive markers of early DN and its fibrotic progression [68], as well as their promise as therapeutic targets for slowing DN progression.

NcRNAs are dynamically regulated in AKI and influence both injury and repair processes. miRNAs: Many miRNAs are acutely altered during AKI and show translational potential as biomarkers of injury severity. For example, urinary miR-30c-5p and miR-192-5p have been shown to rise within 2 h of cardiac surgery-associated AKI, preceding elevations in the classic marker KIM-1 [69]. In sepsis-associated AKI, circulating miR-29a and miR-10a-5p levels correlate with worse 28-day survival [70]. miR-21 is broadly upregulated in AKI and plays context-dependent roles: in septic AKI, miR-21-5p is induced in plasma and kidney tissue and appears to mitigate injury by targeting the pro-apoptotic gene RUNX1, as evidenced by improved renal function and reduced inflammation when miR-21-5p is overexpressed or delivered via endothelial progenitor cell exosomes; indeed, exosomal miR-21 therapy lowered tubular injury in septic AKI models [71]. Conversely, in ischemia–reperfusion AKI, miR-21 has been shown to act as an anti-apoptotic, renoprotective factor (knockdown of miR-21 increased tubular cell apoptosis in preconditioning models). These examples illustrate the dual biomarker and modulatory potential of miRNAs in AKI. For miR-29, temporal dynamics also matter: early after acute injury, transient miR-29 suppression may accompany rapid matrix turnover and repair programs, whereas prolonged loss of miR-29 shifts the balance toward maladaptive fibrosis; restoration of miR-29 activity aligns with resolution of aberrant collagen expression and improved structural recovery [72]. lncRNAs: Dozens of lncRNAs contribute to AKI pathophysiology, particularly in septic injury. Pro-inflammatory lncRNAs such as MALAT1 and NEAT1 are markedly upregulated in endotoxin-induced AKI, where they sponge anti-inflammatory miRNAs (MALAT1 → miR-146a; NEAT1 → miR-204, miR-22-3p), resulting in unchecked NF-κB activation [73,74]. Silencing these lncRNAs in LPS-treated renal cells and septic mouse models reduces NF-κB-driven cytokine release and tubular apoptosis [73]. In contrast, TUG1, HOXA-AS2, and CASC2 are downregulated in AKI, and their overexpression protects by suppressing NF-κB or activating Nrf2-mediated antioxidant pathways [15,75]. Urinary lncRNAs are also candidate biomarkers; TapSAKI is elevated in septic-AKI rats, correlates with tubular injury, and its knockdown alleviates LPS-induced damage in proximal tubule cells [76]. circRNAs: Emerging data implicate circRNAs in AKI. circVMA21 is significantly downregulated in septic AKI, and its restoration alleviates injury by sponging miR-9-3p, thereby upregulating the renoprotective kinase SMG1, reducing apoptosis, oxidative stress, and inflammation [77]. Other circRNAs, including circTLK1 and circPICALM, similarly modulate inflammatory pathways, though research remains preliminary. Collectively, ncRNAs in AKI represent novel early biomarkers (e.g., plasma/urine miRNA panels) and experimental therapeutic targets, where anti-miRs, miRNA mimics, or lncRNA knockdown strategies have shown efficacy in preclinical models [77].

Across both metabolic (diabetic) and acute renal injury, ncRNAs converge on core inflammatory, fibrotic, and cell survival pathways that dictate disease onset and progression. Central regulatory hubs include TGF-β/Smad, NF-κB, and PI3K–AKT/PTEN signaling, alongside apoptosis and extracellular matrix remodeling cascades (e.g., Bcl-2/Bax, collagen I/III/IV). miRNAs such as miR-21, miR-29, and miR-192 act as upstream switches of fibrosis, EMT, and injury response; lncRNAs like MALAT1, NEAT1, XIST, and TUG1 modulate these pathways through transcriptional control or miRNA sponging, whereas circRNAs refine the network by targeting specific miRNA–mRNA axes (e.g., circEIF4G2/miR-218/SERBP1). These ncRNA-driven networks are dynamic, context-dependent, and often cell-type-specific, collectively shaping injury, inflammation, and repair processes in DN and AKI.

## 6. ncRNAs in Chronic and Immune-Mediated Kidney Diseases

In chronic kidney disease (CKD), progressive fibrosis is strongly influenced by ncRNAs. MiRNAs: miR-21 is one of the best-characterized pro-fibrotic miRNAs, consistently upregulated across CKD etiologies. It promotes myofibroblast activation and collagen deposition by repressing antifibrotic targets such as SMAD7 and PTEN [78]. Clinical translation includes the anti-miR-21 oligonucleotide lademirsen, tested in phase 2 Alport syndrome patients, which was well tolerated and additive to ACE inhibitor therapy [79]. Conversely, the miR-29 family, which represses collagen genes, is downregulated in CKD and functions as a critical antifibrotic brake; restoration of miR-29 represses collagen gene networks and attenuates fibrosis in UUO and TGF-β-driven models [80]. Several lncRNAs modulate CKD fibrosis. H19 and MIAT are induced by TGF-β1 and promote fibrogenesis, while Gas5 is downregulated and exerts anti-fibrotic activity [81]. In UUO models, H19 knockdown reduces α-SMA and collagen deposition [82], whereas Gas5 deficiency worsens fibrosis. PVT1, located in a diabetic nephropathy susceptibility locus, drives mesangial expansion and enhances TGF-β signaling, representing another therapeutic target in CKD [83]. High-throughput profiling of urine exosomal RNAs has identified lncRNA and tRNA fragment signatures predictive of early CKD [84]. Circular RNAs are emerging as biomarkers and regulators in CKD. Their stability in biofluids makes them attractive for clinical use. Urinary exosomal hsa_circ_0036649 is decreased in patients with severe fibrosis, correlating inversely with fibrosis scores and eGFR [85]. ROC analysis suggests circ_0036649 could discriminate fibrotic CKD. Functionally, circRNAs such as circACTR2 and circCOL1A1 regulate collagen expression, often by sponging antifibrotic miRNAs, though in vivo work is at an early stage. Plasma lncRNA DKFZP434I0714 has also been linked to cardiovascular mortality in ESRD [86]. Together, these findings highlight ncRNAs as critical mediators of CKD pathology and promising targets for antifibrotic interventions.

Glomerulonephritides, including lupus nephritis (LN) and IgA nephropathy (IgAN), are strongly influenced by ncRNAs. Aberrant ncRNA expression in renal and immune cells drives inflammation and tissue injury. The pro-apoptotic lncRNA-p21 is elevated in peripheral mononuclear cells and urinary sediments of LN patients, correlating with disease activity [87,88]. Within the kidney, RP11-2B6.2 is upregulated and enhances type I interferon signaling by repressing SOCS1; its levels track with intrarenal IFN-I activity and histologic inflammation [87,88]. Another study identified lncRNA lnc-DC (a dendritic cell-associated lncRNA) as elevated in LN, particularly in patients with active nephritis, suggesting involvement in antigen-presenting cell activation [89]. Among circRNAs, circHLA-C was identified as a top upregulated transcript in LN biopsies. Its levels correlated with proteinuria, higher creatinine, and crescent formation [88]. Mechanistically, circHLA-C can act as a sponge for miR-150, a microRNA that modulates immune cell activation. In LN patients, renal circHLA-C expression showed an inverse relationship with miR-150 and is thought to promote pathogenic processes by freeing targets of miR-150 from repression [88]. Consistently, inhibition of miR-150 in mouse models ameliorates lupus kidney injury, supporting the relevance of the circHLA-C/miR-150 axis [90]. Clinically, circulating miR-21 and miR-155 distinguish active LN from inactive disease, and a panel of miR-21, miR-150, and miR-423 improves classification of SLE nephropathy [91,92]. In IgAN, ncRNAs also contribute to pathogenesis. Plasma miR-148a-3p and miR-425-3p are significantly upregulated in patients and inversely correlate with eGFR, linking these molecules to disease severity [93]. These miRNAs are thought to contribute to IgAN pathogenesis by influencing B-cell function and IgA production—notably, miR-148a is known to affect IgA glycosylation, a key abnormality in IgAN [93,94]. Transcriptomic analyses of monocytes further revealed hundreds of dysregulated lncRNAs enriched in innate immune and inflammatory pathways, suggesting they shape immune complex deposition and downstream injury. Urinary exosomal miRNAs are also being explored as noninvasive diagnostic markers [93]. Data are emerging for focal segmental glomerulosclerosis (FSGS) and membranous nephropathy. In FSGS, podocyte-enriched lncRNAs such as LOC105375913 and LOC105374325 are upregulated. LOC105375913 promotes fibrosis by sponging miR-27b and activating Snail, while LOC105374325 induces apoptosis through repression of miR-34c/miR-196 and activation of Bax/Bak [50,95]. Across LN, IgAN, and other glomerulopathies, ncRNAs modulate immune activation, podocyte stability, and matrix accumulation. Their measurable presence in blood and urine positions them as promising biomarkers, while mechanistic insights highlight their potential as therapeutic targets to modulate glomerular inflammation.

Across chronic and immune-mediated kidney diseases, ncRNAs orchestrate fibrotic, inflammatory, and immune signaling networks that drive disease progression. In CKD, miRNAs (e.g., miR-21, miR-29) and lncRNAs (e.g., H19, MIAT, Gas5, PVT1) regulate TGF-β/Smad, PI3K–AKT/PTEN, and collagen synthesis pathways, while circRNAs influence antifibrotic miRNA activity. In glomerulonephritides, including LN and IgAN, ncRNAs modulate interferon signaling, NF-κB activation, immune cell function, and podocyte stability, exemplified by lncRNA-p21, RP11-2B6.2, lnc-DC, and circHLA-C/miR-150 axes. Circulating and urinary ncRNAs repeatedly map onto shared immune–fibrotic cascades, providing mechanistic links between disease activity, immune dysregulation, and structural injury. These interconnected ncRNA networks highlight their potential as integrative biomarkers and therapeutic targets across both chronic fibrotic and immune-driven renal disorders.

## 7. ncRNAs as Biomarkers in Nephrology

**Figure 5:**
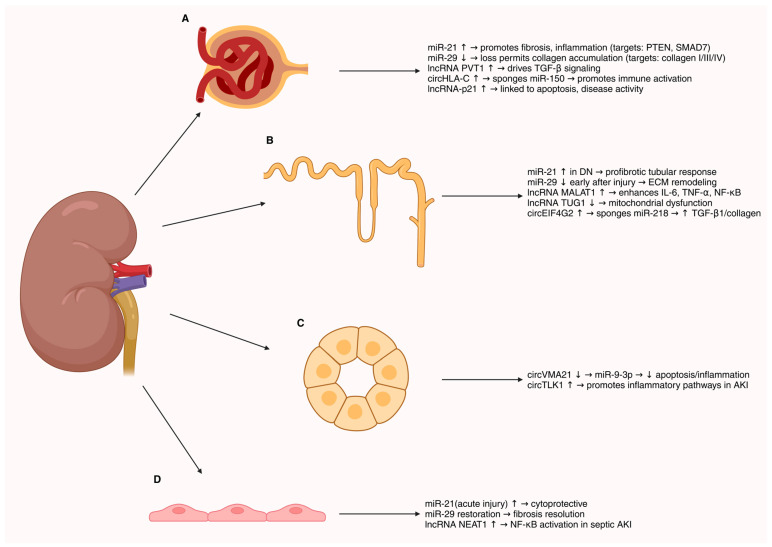
Summary of key ncRNAs and their core targets in the nephron. Representative microRNAs, lncRNAs, and circRNAs with recurrent roles across kidney diseases are mapped to their predominant sites of action: (**A**) glomerulus/podocytes, (**B**) proximal tubule, (**C**) distal tubule/collecting duct, and (**D**) endothelial/interstitial compartments. Arrows indicate regulatory effects (e.g., promotion of fibrosis, immune activation, cytoprotection). This schematic highlights the cell-specific and context-dependent integration of ncRNA networks in renal pathophysiology. Arrows indicate the direction of expression change: upward arrows (↑) represent increased expression, whereas downward arrows (↓) denote decreased expression in kidney disease samples compared with controls.

A crucial field of nephrology is the search for trustworthy biomarkers for kidney disorders to enhance prognosis, early diagnosis, and individualized treatment plans. Because of their exceptional stability in a variety of biofluids and their role in the pathophysiology of many renal illnesses, ncRNAs have become attractive biomarker candidates. lncRNAs, circRNAs, and miRNAs are examples of circulating ncRNAs that can be found in urine, plasma, and exosomes. These circulating ncRNAs offer minimally intrusive windows into kidney health and disease states [96,97,98,99]. Figure 5 provides an overview of the major biological fluids used for ncRNA detection (urine, plasma, serum, exosomes) and the principal analytical platforms applied for their quantification, including qRT-PCR, RNA sequencing, microarrays, and droplet digital PCR. This schematic highlights the methodological diversity underlying ncRNA biomarker studies in nephrology.

Urine is an ideal biofluid for kidney disease biomarker discovery because it directly reflects renal pathophysiology. Several studies have identified specific miRNAs and lncRNAs in urine that correlate with disease presence and severity. For example, urinary levels of the lncRNA GAS5 have been shown to inversely correlate with renal fibrosis severity in CKD, demonstrating superior diagnostic accuracy compared to traditional markers like estimated glomerular filtration rate (eGFR) and TGF-β1 (fibrosis marker) [100,101]. Similarly, dysregulated miRNAs in urine have been linked to diabetic nephropathy and AKI, reflecting processes such as inflammation, tubular damage, and fibrosis [102].

Plasma ncRNAs also provide valuable insights into systemic and renal-specific disease processes [65]. Circulating miRNAs show disease-specific expression patterns in hereditary kidney disorders such as autosomal dominant polycystic kidney disease (ADPKD) and can reflect pathological alterations in cystic growth and renal cell metabolism. Additionally, studies have reported altered plasma levels of certain lncRNAs in diabetic nephropathy, which correlate with disease progression and proteinuria severity, suggesting their utility in monitoring disease status [103,104].

Exosomes and other extracellular vesicles serve as protective carriers for ncRNAs, enabling their stable circulation in biofluids and facilitating intercellular communication. Exosomal miRNAs and lncRNAs derived from renal cells carry disease-relevant molecular signals and have been proposed as robust biomarkers for early kidney injury and chronic disease stages. Their selective packaging in exosomes enhances their stability and specificity, mitigating RNA degradation challenges faced by free circulating RNAs [105,106,107].

NcRNAs exhibit dual potential as diagnostic and prognostic tools in nephrology. Diagnostic biomarkers facilitate early and accurate disease detection, while prognostic biomarkers predict disease trajectory and response to treatments [108,109]. Specific ncRNA signatures have been identified that distinguish patients with kidney diseases from healthy individuals with high sensitivity and specificity. For instance, urinary levels of lncRNA PANDAR are elevated in diabetic patients with significant proteinuria and can predict progression of diabetic nephropathy, serving as both diagnostic and prognostic indicators. Moreover, panels of miRNAs have been developed to differentiate AKI subtypes and predict recovery versus progression to chronic kidney disease after acute injury [101,110,111]. A fundamental advantage of ncRNAs as biomarkers lies in their substantial stability in biofluids. Unlike many proteins that are susceptible to enzymatic degradation, ncRNAs, especially those encapsulated within exosomes or bound to RNA-binding proteins, demonstrate resistance to harsh conditions such as RNase activity, pH variations, and freeze–thaw cycles. This intrinsic stability enables reliable detection, handling, and storage, increasing their suitability for clinical application [112,113,114].

Furthermore, ncRNAs provide tissue- and cell-type specificity, reflecting underlying pathophysiological changes more directly than many circulating proteins, which often have broader expression patterns. This specificity enhances the sensitivity and robustness of ncRNAs as biomarkers for kidney diseases, potentially enabling earlier diagnosis and more precise disease monitoring. Their involvement in key regulatory pathways also opens opportunities for combined biomarker and therapeutic target applications, integrating molecular diagnostics with novel treatment strategies [115,116,117].

In summary, circulating ncRNAs represent a highly promising class of biomarkers in nephrology, offering minimally invasive, stable, and disease-specific molecular indicators. Continued advances in high-throughput sequencing, RNA detection technologies, and validation studies will be essential to translate ncRNA biomarker discoveries into routine clinical practice, ultimately improving patient outcomes in kidney diseases.

## 8. Therapeutic Targeting of ncRNAs

Therapeutically modulating ncRNAs is emerging as a rational strategy to intercept kidney injury and fibrosis at their regulatory roots. Approaches fall broadly into two categories: inhibition of pathogenic miRNAs using antagomirs/anti-miRs with restoration of protective miRNAs using mimics; and silencing of lncRNAs that drive inflammation and fibrosis with siRNAs or antisense oligonucleotides (ASOs). While the renal tract is, in principle, an attractive target for oligonucleotide drugs because phosphorothioate (PS)-modified oligos are avidly taken up by proximal tubules, translation has been gated by delivery, durability, and safety concerns that are only now being addressed [118]. An integrated overview of modalities, mechanisms, and key evidence is summarized in Table 1.

Among pro-fibrotic miRNAs, miR-21 is the most extensively studied in the kidney. Preclinical inhibition of miR-21 reduces tubular EMT, inflammation, and matrix accumulation across models of DN and obstructive nephropathy by derepressing anti-fibrotic pathways (e.g., PTEN/TIMP3), motivating clinical development of anti-miR-21 agents [119]. The first renal disease trial to read out, a randomized controlled study of lademirsen (anti-miR-21) in adults with Alport syndrome at risk of rapid progression, demonstrated acceptable tolerability but failed to improve eGFR versus placebo at weeks 24 or 48, underscoring the challenge of translating robust animal signals into human benefit [79]. Key preclinical and clinical data for anti-miR-21 are listed in Table 1.

Compensating for loss-of-function miRNAs using mimics is the reciprocal strategy. The miR-29 family represses a broad collagen/fibronectin gene network and is consistently downregulated in renal fibrosis. In mice, overexpression or delivery of miR-29 attenuates fibrosis in unilateral ureteral obstruction (UUO) and related models; in DN models, restoring miR-29a/29b limits mesangial expansion and fibrotic signaling. Although most clinical experience with miR-29 mimics (e.g., remlarsen/MRG-201; next-gen MRG-229) is in extra-renal fibrosis (skin, lung), these agents establish proof of mechanism and a growing safety database for systemic miRNA replacement with anti-fibrotic pharmacodynamics that are mechanistically relevant to CKD [64,120]. In practice, miRNA mimics are administered intravenously or subcutaneously as chemically stabilized double-stranded oligonucleotides or in lipid nanoparticles, and they enter cells mainly through receptor-mediated endocytosis followed by partial endosomal escape, which enables the guide strand to be loaded into AGO/RISC [80,121]. See Table 1 for a concise summary of the miR-29 mimic program.

Outside of DN/CKD, miRNA-17 antagonism is being advanced specifically for autosomal dominant polycystic kidney disease (ADPKD). Genetic and pharmacologic data converge on miR-17 as a driver of cyst growth via metabolic and mTOR pathways and direct repression of PKD1/PKD2; kidney-preferring anti-miR-17 oligonucleotides (RGLS4326; next-gen RGLS8429) reduce cystogenesis in multiple mouse models and have progressed to early clinical testing in ADPKD [122,123]. Table 1 summarizes the anti-miR-17 clinical translation.

ncRNAs scaffold chromatin modifiers and transcriptional complexes to enforce sustained inflammatory and fibrotic pathways. In kidney fibrosis, Smad3-associated lncRNAs have emerged as tractable markers. Silencing the Smad3-dependent lncRNA Erbb4-IR restores Smad7 or miR-29 signaling and markedly reduces matrix deposition in UUO and type 2 DN models, highlighting a mechanistically precise way to interrupt TGF-β/Smad3 pathways without globally inhibiting TGF-β. These proof-of-concept studies used siRNA/shRNA or gapmer-style antisense oligonucleotides, which contain a central DNA ‘gap’ flanked by chemically modified RNA-like wings that recruit RNase H to degrade the target RNA, and demonstrate on-target disease modification in the kidney [124,125]. Beyond these, additional lncRNAs (e.g., KCNQ1OT1, IRAR) have been implicated in tubular injury, chemokine induction, and fibrosis, and respond to silencing in preclinical models, supporting the broader therapeutic logic of lncRNA targeting in AKI and CKD [126,127].

PS-modified ASOs and many siRNAs show preferential exposure in the proximal tubule due to receptor-mediated endocytosis, which is an advantage for tubular targets but a challenge for glomerular or interstitial cell types. Nanoparticle formulations, peptide/ligand conjugates, and engineered extracellular vesicles are being developed to broaden cell-type reach in the kidney [128,129]. Seed-mediated off-targeting, defined as unintended binding via the seed region (nucleotides 2–8) to partially complementary sites in non-target transcripts, and unintended pattern-recognition receptor activation remain class risks. Chemical optimization (2′-O-Me/2′-F, constrained sugars, locked nucleic acids (LNAs), and novel backbones such as serinol nucleic acids) and improved sequence selection mitigate these issues, but careful transcriptomic and cytokine profiling is necessary in kidney-directed processes [130]. While most PS-ASO renal effects are low-grade and reversible (e.g., low-molecular-weight proteinuria from competitive inhibition of endocytosis), ASO-associated tubular injury has been observed with certain sequences (e.g., SPC5001), and nephrotoxicity is a leading translational concern for chronic dosing in CKD. Dedicated renal safety pharmacology and biomarkers (KIM-1, clusterin, β2-microglobulin) are therefore integral to development plans [131]. Delivery technologies, chemistries, and safety markers are summarized in Table 1.

Clinical experience in kidney indications is still weak. In Alport syndrome, the phase 2 randomized trial of the anti-miR-21 lademirsen met safety but not efficacy endpoints on eGFR decline at 24–48 weeks [79]. In ADPKD, anti-miR-17 agents progressed from robust preclinical efficacy to human studies: RGLS4326 showed kidney-preferential distribution and biomarker engagement, and the next-generation RGLS8429 is under clinical evaluation (Phase 1b/2 design focused on safety and pharmacodynamic biomarkers such as urinary polycystins). Outcome data are pending [132,133]. A consolidated view of these programs appears in Table 1.

**Table 1:** Therapeutic strategies targeting ncRNAs in kidney disease: mechanisms, key evidence, and representative references.

Therapeutic Strategy	Mechanism/Rationale	Evidence/Key Findings	References
Anti-miR-21 (antagomirs/ASOs)	Inhibition of pro-fibrotic miR-21; derepression of PTEN/TIMP3 and anti-fibrotic pathways	Preclinical: reduced EMT, inflammation, fibrosis in DN/UUO; Clinical (Alport, lademirsen): safe but no eGFR benefit at 24–48 weeks	[79,119]
miR-29 mimics (remlarsen/MRG-201, MRG-229)	Restoration of miR-29 family to repress collagen/fibronectin genes	Preclinical: attenuates fibrosis in UUO and DN; Clinical (skin/lung fibrosis): proof-of-mechanism and safety established, anti-fibrotic PD relevant to CKD	[64,120]
Anti-miR-17 (RGLS4326, RGLS8429)	Blockade of miR-17 to relieve repression of PKD1/PKD2, metabolic/mTOR regulation	Preclinical: reduced cystogenesis in ADPKD models; Clinical: early-phase studies with kidney-preferential distribution, biomarker engagement, safety evaluation ongoing	[122,123,132,133]
lncRNA Erbb4-IR silencing (siRNA/ASO)	Interrupts Smad3-dependent pro-fibrotic signaling; restores Smad7/miR-29 axis	Preclinical: reduces matrix deposition in UUO and type 2 DN models	[124]
lncRNA Arid2-IR silencing (siRNA/ASO)	Inhibition of NF-κB-driven inflammatory pathways	Preclinical: diminished renal inflammation in vivo	[125]
Other lncRNA targets (KCNQ1OT1, IRAR, etc.)	Modulate tubular injury, chemokine induction, and fibrosis	Preclinical: silencing reduces injury/fibrosis in AKI and CKD models	[126,127]
Advanced delivery platforms (nanoparticles, peptide/ligand conjugates, EVs)	Broaden renal cell-type reach beyond proximal tubules	Preclinical: improved biodistribution and efficacy under development	[128,129]
Chemical modifications (2′-O-Me, 2′-F, LNA, novel backbones)	Reduce off-target and immune activation risks	Mitigated toxicity and improved stability demonstrated; translational safety still requires monitoring	[130]
Renal safety biomarkers (KIM-1, clusterin, β2-microglobulin)	Monitor nephrotoxicity and tubular injury during oligonucleotide therapy	Observed low-grade tubular effects; some ASOs (e.g., SPC5001) linked to nephrotoxicity → biomarkers integral for development	[131]

Abbreviations: UUO, unilateral ureteral obstruction; DN, diabetic nephropathy (standardized in text as diabetic kidney disease, DKD); CKD, chronic kidney disease; AKI, acute kidney injury; glomerulonephritis; PKD, polycystic kidney disease; ADPKD, autosomal dominant polycystic kidney disease; eGFR, estimated glomerular filtration rate; EMT, epithelial–mesenchymal transition; PD, pharmacodynamics; mTOR, mechanistic target of rapamycin; ASO, antisense oligonucleotide; siRNA, small interfering RNA; LNA, locked nucleic acid; 2′-O-Me, 2′-O-methyl modification; 2′-F, 2′-fluoro modification; EVs, extracellular vesicles; KIM-1, kidney injury molecule-1.

## 9. Challenges and Future Directions

ncRNAs are essential regulators of renal physiology and play a significant role in the pathogenesis of kidney diseases. They regulate inflammation, fibrosis, apoptosis, and cellular repair, and their unique properties make them promising candidates for biomarkers and therapeutic targets. Despite this potential, translating ncRNA research into clinical practice faces several challenges, which are mainly technical or biological [13,14,15,16].

While technical challenges limit the accurate detection of ncRNAs, their biological complexity further complicates their study. This complexity is due to their context-dependent functions, meaning their effects can vary based on cell type, disease stage, or microenvironment, which makes interpretation and therapeutic use more difficult [134]. Furthermore, multiple ncRNAs can target the same signaling pathways, leading to functional redundancy, as compensatory mechanisms often buffer the effects of altering a single ncRNA [8]. Efficient and specific delivery of ncRNAs to renal cells remains a major challenge, as biological barriers and lack of targeted delivery often limit their therapeutic potential [135]. To address this and better understand ncRNA function, it is important to carefully map their regulatory networks and consider specific disease and cell context. Combining multi-omics data and performing thorough network analyses can help clarify these complex interactions, making it easier to predict ncRNA activity and their potential therapeutic effects [8,136].

In conclusion, translating the biological insights about ncRNAs into clinical applications continues to be challenging. Efficient and specific delivery to renal cells, minimization of side effects, and developing reliable clinical assays represent critical challenges. However, the stability of ncRNAs in biofluids such as urine, plasma, or exosomes makes them particularly promising for non-invasive biomarker applications [96]. Other concerns, including long-term safety, potential immunogenicity, and variability in patient responses, must be resolved before ncRNA-based therapies can be widely implemented [137].

Looking ahead, the integration of ncRNAs into precision nephrology, which aims to tailor diagnosis and treatment to individual patients, offers a promising path toward earlier diagnosis, more accurate disease stratification, and personalized therapeutic strategies [138]. Achieving this will require standardized detection methods, reliable computational tools for multi-omics analysis, and thorough clinical validation. Using molecular profiling together with comprehensive patient data, along with advanced methods for network analysis, biomarker discovery, and predictive modelling, can advance this progress [111]. Close collaboration among researchers, clinicians, and industry is needed to turn these advances into safe and effective clinical tools. Integrating these approaches could enable personalized risk assessment, monitoring of disease progression, and tailored therapeutic strategies [136].

## Data Availability

No new data were created or analyzed in this study. Data sharing is not applicable to this article.
